# Mitogen-Activated Protein Kinase and Nuclear Hormone Receptor Crosstalk in Cancer Immunotherapy

**DOI:** 10.3390/ijms241713661

**Published:** 2023-09-04

**Authors:** Elke Burgermeister

**Affiliations:** Department of Medicine II, Medical Faculty Mannheim, Heidelberg University, Theodor-Kutzer-Ufer 1-3, D-68167 Mannheim, Germany; elke.burgermeister@medma.uni-heidelberg.de; Tel.: +49-621-383-2900

**Keywords:** MAPK, kinase, nuclear receptor, hormone, cancer, immunotherapy

## Abstract

The three major MAP-kinase (MAPK) pathways, ERK1/2, p38 and JNK/SAPK, are upstream regulators of the nuclear “hormone” receptor superfamily (NHRSF), with a prime example given by the estrogen receptor in breast cancer. These ligand-activated transcription factors exert non-genomic and genomic functions, where they are either post-translationally modified by phosphorylation or directly interact with components of the MAPK pathways, events that govern their transcriptional activity towards target genes involved in cell differentiation, proliferation, metabolism and host immunity. This molecular crosstalk takes place not only in normal epithelial or tumor cells, but also in a plethora of immune cells from the adaptive and innate immune system in the tumor–stroma tissue microenvironment. Thus, the drugability of both the MAPK and the NHRSF pathways suggests potential for intervention therapies, especially for cancer immunotherapy. This review summarizes the existing literature covering the expression and function of NHRSF subclasses in human tumors, both solid and leukemias, and their effects in combination with current clinically approved therapeutics against immune checkpoint molecules (e.g., PD1).

## 1. Introduction

MAP-kinases (MAPKs) and their upstream tyrosine kinase receptors (RTKs) of the epidermal growth factor receptor (EGFR/*Her*) family are major drug targets in clinical oncology [1]. However, current clinical therapies are still limited in success due to largely unknown resistance mechanisms in cancer patients. The recent breakthrough in immunotherapies is based on the strategy to exploit the host immune system to recognize and eliminate tumor cells with aberrant RTK signaling [2]. Antibodies (Abs) targeting regulatory immune checkpoint molecules (e.g., PD1, PDL1, CTLA4) on lymphocytes, mainly T-cells, and antigen-presenting cells including dendritic cells (DCs) and macrophages, reinforce the host’s anti-tumor response and reshape the immunosuppressive tumor tissue microenvironment into a permissive mode towards combination therapies including small molecules and adoptive cell transfer (e.g., CAR-T cells [3]). Since only a subset of patients with hypermutated (microsatellite instable) tumors are currently eligible for immunotherapy [4], the identification and validation of sensitizer drugs are of medical need to include larger patient groups and improve clinical outcomes. 

The nuclear hormone receptor superfamily (NHRSF) comprises at least 48 members, many of them drugable by agonists or antagonists that are either in preclinical development or already in long-term clinical use (such as steroid analogs) [5]. The main subclasses are classical endocrine hormones of the female and male reproductive tract and peripheral organs (thyroid, kidney, adrenals, bone), metabolic receptors sensitive to dietary input (e.g., lipids, vitamin A/D, xenobiotics) and developmental regulators (“orphan” receptors) with ligands still to be characterized. NHRs are transcription factors of a modular structure with DNA-binding (DBD) and ligand-binding (LBD) domains [6]. They recognize and bind responsive elements in the DNA as monomers, homo- or hetero-dimers, and interact with coregulators (coactivators, corepressors) to modulate the chromatin structure at gene promoters/enhancers relevant in tissue homeostasis, differentiation, metabolism and host immunity. Both receptors and their upstream coregulators interact with and/or are phosphorylated by oncogenic signaling cascades emanating from RTKs at plasma- or endomembranes and downstream MAPK pathways. These post-translational modifications impact both genomic and non-genomic activities of the NHR in either a synergistic (e.g., ER) or antagonistic (e.g., PPARγ) manner.

Therefore, the combination of clinically in-use kinase inhibitors (e.g., against the EGFR, BRAF or MEK1/2) with NHR ligands (Table 1) is a promising avenue for dual targeting of oncogenic signaling in tumor cells and simultaneous empowerment of host immunity. Here, I summarize the current knowledge on pre/clinical efficacies and molecular mechanisms of NHR and MAPK crosstalk with respect to cancer immunotherapy. In the future, an in-depth understanding of these signaling interactions may allow the development of tailored combination strategies for the benefit of cancer patients. 

## 2. General Modes of Action of MAPK and NHR Crosstalk

**Figure 1:**
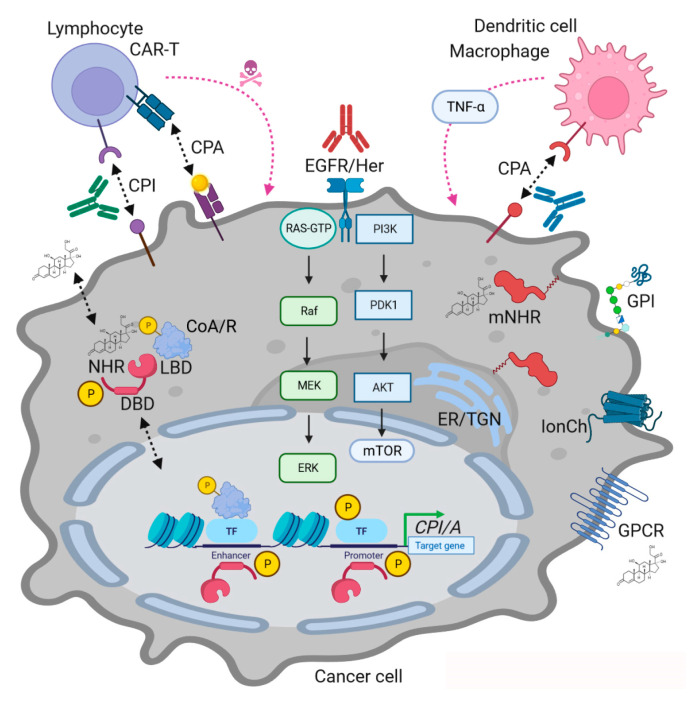
MAPK and NHR interactions in cancer immunotherapy showcasing modes of actions (MOAs) of NHR ligands. Legend (numbered): (a) Genomic MOA: Ligands bind to intracellular NHRs together with coactivators (CoA) or corepressors (CoR) followed by binding to DNA-response elements in promoters or enhancers of target genes to activate or repress transcription in cooperation with other transcription factors (TFs, e.g., NFκB), respectively. MAPKs (and other kinases) phosphorylate (“P”) NHRs, coregulators and transcription factors to fine-tune transcriptional events. (b) Non-Genomic MOA: Ligands bind to membrane-bound intracellular NHRs (mNHR) or to alternative receptors (e.g., GPCRs, GPI-anchored protein, ion channel (IonCh)) or transactivate RTKs (e.g., EGFR family) triggering signal transduction cascades which culminate in phosphorylation of transcription factors or change cell phenotypes independently of transcription (e.g., mitochondria, vesicle traffic, cytoskeleton, etc.). Similar processes occur in immune cells, exemplified here by lymphocytes (T-cells) and antigen-presenting cells (macrophages, dendritic cells). NHR ligands and their receptors alter the expression of inhibitory (CPI) and activatory (CPA) immune checkpoint genes (e.g., PD1, PDL1, CTLA4) and soluble factors (e.g., chemo/cytokines). Pharmacological and genetic intervention with RTK-MAPK signaling by blocking Abs (e.g., EGFR family) or NHR agonists/antagonists (see Table 1) can enhance the recognition, killing and elimination of tumor cells by immune cells (e.g., antibody-dependent cytotoxicity or phagocytosis). Legend: ER/TGN = endoplasmic reticulum and trans-Golgi network.

NHRs exert an array of downstream modes of action (MOAs) translating input from the macro- and microenvironments towards intracellular compartments (Figure 1). Overall, their DBD allows them to reside in or translocate to the nucleus to either repress or induce gene transcription. This action was termed (slow) “genomic” and can comprise interaction with other DNA-bound factors (e.g., NFκB) leading to transactivation or trans-repression, respectively. In addition, (rapid) “non-genomic” effects summarize both nuclear receptor-dependent and ligand-dependent (“hormone-only”) actions. The former comprise interactions of the NHR with cellular signaling (MAPKs, PI3K, etc.) or structural components (e.g., organelles, enzymes, cytoskeleton, vesicles, etc.), while the latter are transduced by membrane-bound non-NHR receptors (e.g., ion channels, transporters, G protein-coupled receptors (GPCRs) or RTKs of the EGFR family). Caveolae, lipid raft and fatty acid-anchored NHRs and non-NHR steroid receptors have been identified and functionally validated. Ligands can also diffuse into the cell interior and directly target intracellular proteins [7,8].

Conclusively, the interplay of both modes of action contributes to the overall outcomes in a given target cell. Thus, intelligent design of drug combinations simultaneously addressing MAPKs and NHRs is a promising approach to co-target tumor and immune cells.

## 3. Endocrine Receptors

### 3.1. Estrogen Receptors (ERs)

ERs (α/β) are prime oncogenic targets for the treatment of ER+HER2- breast cancer, besides their physiological roles in the female reproductive tract and body homeostasis such as the prevention of osteoporosis. Selective ER modulators (SERMs) complement classical ER antagonists (e.g., tamoxifen, raloxifen), whereas aromatase inhibitors restrict ligand availability. ER+PR+HER2+ breast cancers are responsive to anti-hormonal therapy and inhibitory antibodies against the RTK (e.g., trastuzumab), whereas triple-negative cases suffer from poor outcomes due to a lack of efficient therapies and response biomarkers [9]. Thus, combination immunotherapies for breast cancer subtypes are under investigation.

A phase 2 clinical trial (NCT02395627) [10] evaluated patients with ER+ breast cancer upon treatment with histone deacetylase (HDAC) inhibitor (vorinostat), ER antagonist (tamoxifen) and PD1-blocking Ab (pembrolizumab). All responders showed T-cell exhaustion (CD8+ PD1+ CTLA4+) and depletion of regulatory T-cells (Tregs) (CD4+ FOXP3+ CTLA4+), proposing a positive predictive immune signature in PDL1-ER+ patients for response to checkpoint immunotherapy. In a phase 1/2 study (NCT02778685) with cyclin-dependent kinase (CDK) inhibitor (palbociclib), pembrolizumab and depletion of ER ligand by an aromatase inhibitor (letrozole) [11], a complete response rate in patients with ER+HER2- metastatic breast cancer was achieved, warranting confirmatory trials for this clinical benefit. The phase 1b open-label, multicohort KEYNOTE-028 (NCT02054806) study corroborated the safety and anti-tumor efficacy of PD1 Ab (pembrolizumab) in ER+HER2- patients with PDL1+ tumors and prior endocrine therapies [12].

Ex vivo translational studies employing a perfusion bioreactor for tissue engineering to model the tumor microenvironment of freshly excised human breast cancer biopsies evinced therapeutic effects of anti-estrogens, pertuzumab and checkpoint inhibitors on ER+HER2+ tumor cells [13]. Notably, PDL1 and CTLA4 Abs increased the proliferation of lymphocytes, tumor cell death and the synthesis of pro-inflammatory IFNγ at the expense of anti-inflammatory IL10 in triple-negative samples as well.

Constitutive active *ESR1* (ERα) gene mutations are frequent in human metastatic breast cancer [14] and confer resistance to adjuvant therapy with aromatase inhibitors. Wildtype (wt) ER and PR were down-regulated in metastases positive for mutant ER, but accumulated Tregs and helper (Th) T-cells, macrophages and PDL1+ myeloid-derived suppressor cells (MDSCs). Cells or patient-derived xenograft (PDX) models with CRISPR/Cas9-edited mutant ER expressed higher levels of AR, CHI3L1 and IFN-stimulated genes. Notably, AR inhibition abrogated the growth advantage of ER mutant cells to survive estrogen deprivation. Therefore, a combination of AR antagonists with ER degraders (e.g., fulvestrant) and checkpoint blockage may help to foster the clinical response of patients with mutant ER.

The two ER subtypes (α/β) have non-redundant preclinical functions in tumor cells [15], also with regard to specific tyrosine phosphorylation sites. *Esr2* Y55F loss-of-function mice subjected to ERβ agonist S-equol and PD1 Ab displayed a higher tumor burden compared with wt littermates. Mechanistically, ERβ phosphorylation and non-genomic signaling of S-equol in cytotoxic CD8+ effector T-cells (Teffs) boosted T-cell receptor activity and PD1 Ab-driven anti-tumor response.

This finding was reproduced in mouse tumor models under ERβ agonist (LY500307) and PD1 Ab treatment, wherein MDSCs were reduced and infiltration by cytotoxic CD8+ Teffs increased [16]. Tumor cells secreted CSF1/M-CSF which acted as a chemoattractant for CSF1R+ MDSCs in vitro. Notably, the blockade of CSF1R reached comparable anti-tumor efficacy as compared to ERβ agonism in vivo.

Thus, ER-subtype-selective ligands, specifically ERα antagonists as opposed to ERβ agonists, may be suitable to improve the efficacy of checkpoint blockage. Combination with kinase inhibitors, either of RTKs (e.g., HER2, EGFR) or downstream kinases (MAPKs, BRAF, PI3K, CDKs), may be a viable strategy for future clinical applications.

In this context, direct genomic actions of ERs on genes encoding for checkpoint molecules are likely to contribute to this mechanistic crosstalk. For example, ERα down-regulated *PDL1* gene transcription in human breast cancer cells in vitro [17]. In contrast, estrogen [17β-estradiol (E2)] increased intracellular PD1 protein and promoted immunosuppression mediated by CD4+FOXP3+ Tregs, and, consistently, PD1 and immunosuppression by Tregs were reduced in ER knockout mice [18]. Likewise, estrogen post-transcriptionally stabilized *PDL1* mRNA via PI3K-AKT signaling and in an ER-dependent manner in human breast cancer cells [19].

Multiple non-genomic mechanisms have been documented for steroids. As such, estrogen signaling via G-protein-coupled-estrogen-receptor-1 (GPER1/GPR30) elicits the infiltration of inflammatory macrophages and exclusion of FOXP3+ Tregs from tissues by the up-regulation of MCP1/CCL3 and endothelin-1 in mice [20]. GPER [21] activation also promoted the differentiation of primary melanocytes and melanoma cells in vitro. In tumor-bearing mice, GPER1 agonist (G-1) prolonged survival in combination with PD1-blocking Ab. In human gastric cancer cells/tissues and mouse xenografts, G-1/GPER1 signaling [22] provoked endoplasmic reticulum stress and apoptosis, and [23] G-1 inhibited endotoxin-mediated expression of pro-inflammatory cytokines (TNFα, IL6, IFNγ, IL17) in human and mouse macrophages independently of Erα/β, thereby ameliorating symptoms in mice with experimental autoimmune encephalomyelitis (EAE), a surrogate of human multiple sclerosis.

Since GPCRs next to classical second messenger signaling systems also employ downstream MAPK signaling pathways, this crosstalk of NHR ligands and non-NHR receptors may be an interesting avenue for future immunotherapy combinations.

Ample evidence exists for ERs and their coactivators (e.g., SRC3) to be direct targets for serine/threonine-directed phosphorylation by MAPKs [24], with only a few reports on the impact of combined inhibition of both MAPK pathways and immune checkpoints. 

An elegant study demonstrated that [25] anti-estrogen fulvestrant and pan-HER inhibitor (dacomitinib) reduced the oncogenic interplay between ER and RTKs (EGFR) to exert synergistic anti-tumor effects in mouse models of *KRAS* mutant lung cancer. In syngeneic and xenograft models, both drugs induced an inflamed tumor tissue microenvironment with increased numbers of myeloid cells, cytotoxic CD8+ Teffs and PD1 expression. Consequently, sequential triple therapy with PD1 Ab following the two drugs potentiated tumor growth inhibition in these mice.

Likewise, CDK4/6 inhibitor (palbociclib) enforced the anti-tumor response in patients with metastatic ER+ breast cancer [26]. To prevent relapse, BCL2 inhibitor ABT199 (venetoclax), in combination with ER antagonist (fulvestrant) and palbociclib, was administered to ER+ breast cancer cell lines, patient-derived organoid (PDO), PDX and syngeneic immunocompetent mouse models. Triple therapy effectively triggered cell cycle arrest, apoptosis and senescence (senolysis) in tumor cells, and together with PD1 Ab reshaped the tumor microenvironment to a favorable immune profile.

In summary, intervention at the level of the ER subtypes or upstream at the level of ER-ligand availability holds promise for the effective treatment of molecular breast cancer subtypes in combination with kinase and immune checkpoint inhibitors. 

### 3.2. Progesterone Receptor (PR)

PR is the second most relevant predictive biomarker for hormone-dependent breast cancer and is used for diagnostics with ER and HER2 in tissue specimens. However, selective PR antagonists (e.g., mifepristone (RU486)) are currently prescribed only for medical abortion and miscarriages in women. Hitherto, selective PR modulators (SPRMs) have not been approved for clinical cancer treatment, and their utility in combination with immunotherapies remains elusive.

A recent clinical case report [27] from an investigator-initiated study claimed that mifepristone as a single agent prevented the progression of stage IV PDL1+ non-small-cell lung cancer in a patient who failed to respond to prior chemotherapy and PD1-blocking Ab (nivolumab).

A preclinical study found [28] that mifepristone exerts anti-tumor efficacy against hormone-dependent breast cancer in mice via inhibition of tumor growth and reprogramming of the tumor microenvironment. Pro-inflammatory chemokines and cytokines, infiltration of macrophages, natural killer (NK) cells and central memory CD8+ T-cells as well as DC maturation were increased, and the presence of alarmins (calreticulin, high mobility group box 1 protein HMGB1) indicated immunogenic cell death. Mifepristone sensitized PR+ tumors to blockage by PDL1 Ab in this in vivo model.

Overall, anti-progestins are expected to improve the success of checkpoint immunotherapies in PR+ tumors; nonetheless, further preclinical studies are necessary to determine the safety profile.

Despite the lack of experimental evidence for a direct genomic action of PR on immune checkpoint expression, non-genomic crosstalk with MAPK pathways has been reported in endometrial cancer [29]. Double-mutant mice with deletion of tumor suppressor PTEN and overexpression of oncogenic *KRAS* (*PR*(*cre*/+)*Pten*(*f*/*f*)*Kras*(*G12D*) had severe tumor burden and more invasive cancers than littermates with single mutations. PR expression was down-regulated followed by de-repression of ER signaling, confirming the impact of RAS/PI3K-pathway-driven NHR modulation on endometrial cancer progression. 

Bona fide N-terminal phosphorylation sites in steroid receptors are targeted by proline-directed kinases of the MAPK and CDK families [30], generating a second signal for input cues other than NHR ligands. As for estrogens, progesterones also recognize non-NHR receptors to evoke rapid signaling. Membrane-associated-progestin-and-adipoQ-receptors (mPRs/PAQR5-9) [31] activate JNK1/2 and p38 MAPKs to elicit apoptosis in ovarian cancer cells, and progesterone receptor membrane component 1 (PGRMC1) augments breast cancer growth via transactivation of EGFR-PI3K-AKT signaling [32]. Moreover, activation of mPRα by albumin-conjugated progesterone induced the expression of pro-inflammatory genes (COX2, IL1β, TNFα) via activation of PKA-CREB-MEK1/2 signaling in the murine macrophage cell line (RAW264.7) [33]. Hence, deciphering this molecular crosstalk between ER/PR signaling and drugable MAPK pathways with regard to immunotherapies is pending, awaiting validation in preclinical and clinical studies.

### 3.3. Androgen Receptor (AR)

Androgen deprivation therapy by anti-androgens (e.g., abiraterone, enzalutamide, darolutamide, etc.) enjoys a long history of clinical success in patients with hormone-sensitive prostate cancer. However, tumors refractory to castration or hormonal intervention remain a major obstacle to improving patient survival (Clinical Trials: [34,35,36]). Immunotherapy retains promising options although prostate cancers are “cold” tumors with low immune cell infiltration.

Spatial profiling of the transcriptome and proteome from ex vivo clinical metastases evinced high variation in AR and neuroendocrine activity, and the absence of inflammatory infiltrates and immune checkpoints (PD1/PDL1/CTLA4) [37]. Nonetheless, AR expression correlated with B7-H3/CD276, a potential novel targetable immune checkpoint that remains to be verified in preclinical studies.

So far, immunotherapy in patients with advanced prostate cancer has failed [38]. However, a landmark preclinical study evinced that AR blockade sensitized tumor-bearing mice to PD1 Abs by enhancing the function of cytotoxic CD8+ T-cells. AR inhibition prevented T-cell exhaustion via up-regulation of *Ifng* gene expression. Thus, interference with T-cell intrinsic AR functions allows the development of novel strategies to break resistance to immunotherapy. 

In contrast, high-dose dihydrotestosterone abrogated the cytotoxicity of NK cells towards castration-resistant prostate cancer cells via AR/circFKBP5/miRNA-513a-5p/PDL1 signaling. In mouse xenograft models [39], PDL1-blocking Ab or shRNA knockdown synergized with the hormone to diminish tumor cell growth, claiming that the combination of high-dose androgen with PD1/PDL1 inhibitors may ameliorate cancer immunosurveillance.

Patients with metastatic castration-resistant prostate cancer develop de novo resistance to checkpoint blockade mediated by MDSCs which support immune evasion [40]. In an autochthonous, chimeric mouse model, CTLA4 or PD1 Abs achieved robust synergistic responses when combined with multi-kinase inhibitors (cabozantinib, BEZ235). This treatment suppressed the production of IL1RA and cytokines which nourish the development and recruitment of MDSCs. Thus, immune checkpoint blockade together with MDSC-targeted therapy may improve clinical outcomes in patients.

Interestingly, CD8+ T-cells underlie gender-related differences regarding adaptive anti-tumor immunity [41]. As such, AR lowered both activity and stemness of tumor-infiltrating CD8+ T-cells in male mice, and castration combined with PDL1 Ab synergistically dampened tumor growth. Similar results were observed in patients, where CD8+ T-cells from male individuals failed to remain in a stem cell-like memory state and displayed more AR-driven exhaustion phenotypes than female ones. Thus, gender-selective administration of endocrine and immunotherapeutics shall be mandatory to guarantee optimal individualized treatment for each patient.

Direct genomic actions of the AR on checkpoint genes are known. In human hepatocellular carcinoma cells, AR boosts the response to immune checkpoint therapy by down-regulating PDL1 [42]. AR directly repressed transcription of the *PDL1* gene and enhanced CD8+ T-cell functions in vitro. In mice, reduced AR expression yielded improved response for PDL1 Ab, a finding to be translated into patients to explain gender disparity in clinical outcomes during immunotherapies.

Overexpression of B7H3 is a negative prognostic factor and potential immune checkpoint target in prostate cancer [43]. Enzalutamide-resistant B7H3+ human metastatic prostate cancer cells exhibited a strong signaling signature for AR itself and its co-factors (HOXB13, FOXA1) which directly bound and transactivated the promoter and distal enhancer regions of the *B7H3* gene, proposing this B7 family protein as a target for inhibitory Abs.

Despite the plethora of knowledge regarding the phosphorylation of AR and its coactivators (e.g., by MAPKs, CDKs) in prostate cancer [44,45,46], only limited evidence exists for non-genomic crosstalk of AR with kinases.

In mice with syngeneic prostate cancer [47], knockdown or pharmacological inhibition of p21-activated kinase-4 (PAK4) sensitized to PD1 Ab. PAK4 expression was controlled by AR and FOXO1 transcription factors and associated with low intra-tumoral immune cell counts, whereas PAK4 loss increased CD8+ T-cell infiltration, expression of IFNγ response genes and endothelial cell adhesion molecules for improved vascularization and chemotaxis of immune cells into the tumor microenvironment.

A clinical study corroborated that AR blockade promotes response to neoadjuvant BRAF/MEK-targeted therapy and increases recurrence-free survival in melanoma patients (NCT02231775) in a gender-specific manner [48]. Improved progression-free and overall survival prevailed preferentially in female patients. Accordingly, impaired anti-tumor activity was stated in male mice with elevated AR levels upon BRAF and MEK1/2 inhibition compared to female ones. Nonetheless, pharmacological AR inhibitors improved, whereas AR agonism (testosterone) impaired response to BRAF/MEK-targeted therapy in patients of both genders.

Similar to other steroids, non-genomic signaling of testosterone occurs via membrane-bound non-NHR receptors [49,50], which may explain the anti-tumoral effects of supra-physiological testosterone (bipolar androgen therapy) vs. androgen deprivation in castration-resistant prostate cancer. Non-NHR receptors in human prostate cancer cells are exemplified by the GPCR oxoeicosanoid receptor 1 (OXER1), where androgen antagonizes with 5-OxoETE for the rapid reorganization of the actin cytoskeleton promoting migration and metastasis [51]. GPRC6A can transduce testosterone signaling towards ERK1/2-p70S6 kinase and mTORC1 [52], resulting in cell proliferation and the inhibition of autophagy. ZIP9 (SLC39A) [53] is a zinc transporter suggested to bind androgens triggering G protein-coupled ERK1/2 activation and zinc flux, although ion channels and their role in steroid signaling remain to be specified.

Conclusively, tailored targeting of sex/gender-selective steroid receptors in combination with kinase pathway inhibitors and immunotherapeutics shall be considered for future regimens of precision oncology. The relevance of membrane-associated AR, PR, or ER variants or equivalent receptors (e.g., GPCRs, RTKs) transducing rapid, non-genomic signaling has yet to be verified.

### 3.4. Glucocorticoid Receptor (GR)

Corticosteroids (e.g., (hydro)cortisone, prednisone/prednisolone, dexamethasone), pharmacological derivatives of the physiological stress hormone cortisol, are widely prescribed anti-inflammatory and immunoregulatory GR agonists. 

GR targeting for cancer immunotherapy has been recently appreciated in the prevention of immune-related adverse events (IRAE) under checkpoint therapy [54]. “Un-braking” physiological tolerance by PD1/CTLA4 Abs unleashes auto-immunity against normal tissues presenting as “cytokine storm” which manifests as local-to-systemic inflammation (colitis, hepatitis, pneumonia, myocarditis, etc.) and damage of endocrine organs (diabetes, hypothyroidism). Thus, topical or systemic GR agonists are administered to restore the balance between effective anti-tumor immunity and self-tolerance to normal tissue.

The phase 3 clinical trial (NCT03617731) succeeded in testing combinations of blocking Abs against CD38 on plasma cells (isatuximab) with global protein-degrading agents (lenalidomide, bortezomib) and dexamethasone in patients with multiple myeloma [55]. A mechanism-based study (NCT02336815) evinced an improved response in multi-refractory multiple myeloma by providing dexamethasone and selinexor [56], an inhibitor of exportin-1 (CRM1) which traps active tumor suppressor proteins in the nucleus and prevents NFκB activation and the translation of oncoprotein mRNAs. A landmark report explored the influence of psychological stress on the GR-TSC22D3 axis in anti-tumor immunity [57]. In patients, corticosterone up-regulated TSC22D3, which diminished type I/II IFN responses in DCs and T-cells. Vice versa, injection of GR antagonist or *Tsc22d3* deletion in mice rescued anti-cancer immunosurveillance.

The most prominent gene addressed as a checkpoint receptor/ligand system is “glucocorticoid-induced-tumor-necrosis-factor-receptor-related-protein” (TNFRSF18/GITR). To boost preclinical and clinical efficacy via receptor clustering, an anti-PD1-GITRL bispecific agonist has been developed for optimized T-cell receptor activation, proliferation and generation of PD1+GITR+ memory T-cells [58]. This Ab mitigated tumor growth in genetic, syngeneic and humanized xenograft mouse models, encouraging clinical testing [59]. In a phase 1 trial with GITR Ab TRX518 (NCT01239134) as monotherapy, reduced Tregs vs. increased Teffs levels were observed; however, cytolytic T-cells were induced only in combination with PD1 blockage (NCT02628574). 

Lucitanib, a multi-kinase inhibitor, increased CD8+CD4+ T-cell counts and decreased the numbers of DCs and MDSCs in syngeneic mouse colon cancer models [60]. In combination with blocking Abs against inhibitory (PD1, CTLA4) and agonists against activatory (GITR, 4-1BB, ICOS, OX40) immune checkpoints, the anti-tumor response was potentiated. Specifically, intra-tumoral vessel density and macrophage counts were diminished, while CD8+ T-cell infiltration was augmented. Overall, neoangiogenesis and immune signatures of the tumor microenvironment were rewired in a beneficial manner.

GR exploits genomic “trans-repression” as a major mechanism to inhibit transcription factors bound in close proximity to the DNA of common target genes. Direct genomic effects of GR on immune checkpoint genes may interfere with given therapies targeting receptors at the cell surface. As such, GR induced PDL1 and repressed MHC class I gene transcription in pancreatic ductal adenocarcinoma cells [61], and this reciprocal effect was also evident in patients. In contrast, depletion or inhibition of GR in mice reduced PDL1 and restored MHC class I levels, allowing cytotoxic T-cells to infiltrate tumors and overcome resistance to checkpoint inhibitor therapies. 

Natural and synthetic glucocorticoids [62] increased PD1 on T-cells by GR transactivation of a glucocorticoid response element upstream of the transcriptional start site of the *Pdcd1* gene in vitro and in vivo. Thus, PD1-driven T-cell inactivation supports the anti-inflammatory and immunosuppressive actions of glucocorticoids.

Stress-driven GR also up-regulated the immune checkpoint TIGIT on NK and T-cells, including invariant NK (iNKT) and mucosa-associated invariant T (MAIT) cells, to dampen host defense [63]. TIGIT induction was prevented by the GR antagonist RU486. T-cell-specific GR deletion and exposure of mice to stress or oral corticosterone proved that this cell-intrinsic mechanism also exists in vivo. This hormone induced TIGIT, but not CTLA4 or LAG3, on mouse hybridomas and primary cells from explanted organs in a GR-dependent manner.

Thus, direct targeting of the GR may open novel options to counteract adverse and foster verum effects of checkpoint immunotherapies, especially in the presence of physical or psychological stress conditions.

As for all steroid receptors, GR is a major target for phosphorylation by serine/threonine kinases [64], although the relevance of this specific post-translational modification with regard to immunotherapies is still unknown. In addition, non-genomic effects exploiting GR-kinase crosstalk contribute to the potential of GR targeting for the management of cancer, inflammation and autoimmunity.

A landmark study investigated the direct interaction of RAS proteins with the GR [65]. To decipher the mechanism of how GR inhibits PI3K-AKT and MAPK (MEK1/2, p38) signaling, the authors found that apo-GR colocalizes and interacts with wt and mutant K-RAS in the cytoplasm of mouse embryonic fibroblasts and human lung cancer cells. It inhibited RAS activity via the DBD, whereas ligand stimulation or genetic deletion of the GR abolished this effect and exacerbated tumor growth in mice. Accordingly, loss of GR in patients with non-small-cell lung cancer predicted poor prognosis. Therefore, careful use of glucocorticoid is required due to its potential to interfere with K-RAS, a major oncogenic driver of solid tumors with adverse outcomes (e.g., pancreas, colon, lung).

A similar approach was taken by dual targeting of GR and MEK1/2 [66] in mice with RAS-mutated multiple myeloma. Notably, dexamethasone in combination with trametinib acted synergistically against *KRASG12A* mutant cell lines, abolished IGF1-PDK1 survival signaling and triggered apoptotic cell death. This effect was reproduced in xenograft models, proposing novel cures against relapsed and/or refractory disease also for patients.

The add-on benefit of dual targeting was confirmed by the inhibition of RTKs (e.g., EGFR) in non-small-cell lung cancer cells [67]. Here, TNFα inhibitor (etanercept), thalidomide and GR agonist (prednisone) were tested for their ability to counteract compensatory resistance mechanisms induced by RTK inhibitors such as up-regulation of TNF expression. Therein, GR agonism blunted key resistance factors downstream of RTK signaling (e.g., STAT3, YAP, TNF, NFκB). Prednisone combined with EGFR inhibition suppressed adaptive resistance also in vivo (e.g., in mouse models of lung cancer), proposing novel treatment concepts for the human disease.

Hence, the inhibitory action of the GR towards pro-oncogenic and pro-inflammatory kinase signaling is a pivotal avenue to be followed to prevent over-reactions and counterbalance signal activities both in epithelial/tumor and stroma/immune microenvironments. 

Plasma membrane-associated non-genomic GR variants or receptors (e.g., GPCR) are hitherto unknown. They remain to be identified to explain the rapid effects of glucocorticoids on acute inflammatory signaling (e.g., allergic asthma) [68].

### 3.5. Mineralcorticoid Receptor (MR)

MR, also termed aldosterone receptor, is causal for the pathogenesis of kidney disease through aberrant ion excretion and homeostasis disruption, resulting in hypertension and cardiovascular complications. MR antagonists (e.g., spironolactone, finerenone) have been in clinical use for decades to treat patients with hyperaldosteronism.

Recently, finerenone received clinical attention due to its anti-inflammatory protective effects in COVID-19 patients [69]. Thus, one may envision MR repurposing for cancer immunotherapies. 

A clinical trial elaborated that spironolactone lowers blood pressure and plasma concentrations of IFNγ and IL6 in patients with diabetes and hypertension [70]. In vitro, spironolactone reduced endotoxin-stimulated synthesis of pro-inflammatory cytokines (TNFα, IL6, IL1β) in macrophages.

In a preclinical study, aldosterone led to the generation of DC-mediated Th17 type CD4+CD8+ T-cells [71]. The mice displayed MR-dependent activation of MAPKs and production of cytokines (IL6, TGFβ), promoting inflammation and autoimmunity in the context of EAE. In contrast, MR inhibition mitigated these phenotypes, underscoring the findings in humans.

Aldosterone also impairs the function of mitochondria. In human umbilical vein endothelial cells (HUVECs), MR reduced mitochondrial DNA contents and SOD2 expression [72], proposing MR blockage to enforce the detoxification of reactive oxygen species and prevent endothelial dysfunction, a hallmark of tumor diseases. 

In its genomic mode of action, aldosterone-activated MR transcriptionally up-regulated expression of MMP9/NGAL via PI3K and MAPK (p38, ERK1/2) pathways in human neutrophils [73], thereby contributing to extracellular matrix degradation and tissue injury, whereas MR antagonist (spironolactone) abolished this detrimental effect and may thus favor tissue repair.

Likewise, the KRAS4A oncogenic variant was targetable by MR in renal cancer cell lines [74]. Notably, *KRAS* expression was down-regulated by spironolactone, but up-regulated by aldosterone, reciprocally affecting growth and survival signaling via AKT and RAF pathways.

As for other endocrine receptors, phosphorylation [75] by kinases (e.g., ULK1 [76]) and colocalization of MR with and non-genomic activation of the EGFR at cholesterol-rich plasma membrane regions has been demonstrated [77]. Only a small fraction of apo-MR is associated with the cell membrane, while ligand stimulation triggered nuclear translocation and release from the EGFR, followed by its transactivation. In this context, it was further proposed that [78] subcellular trafficking of MR via β-arrestins leads to internalization of the MR and endomembrane-associated signal propagation.

Overall, MR antagonism holds promise to interfere with cancer-relevant pathways such as tissue remodeling, proliferation and angiogenesis. Like for the GR, membrane-associated protein variants of this NHR or equivalent non-genomic receptors are yet to be discovered. 

### 3.6. Thyroid Hormone Receptors (TRs)

Dysfunction of the thyroids is a prevalent disease among the general population worldwide, and thyroid cancers are a major health burden. Inflammation and damage to the thyroid glands, as part of the IRAE spectrum, accompany immune checkpoint therapies in cancer patients [54]. Therefore, targeting TRs (α/β) represents a clinical challenge.

Immune checkpoint inhibitor-related thyroid dysfunction [79] manifests clinically as destructive thyroiditis by cytotoxic T-cells or hypothyroidism and is an obstacle to successful cancer treatment, with patients requiring hormone replacement therapy (e.g., using levothyroxine).

A retrospective study on cancer patients under PD1 Ab therapy (nivolumab, pembrolizumab) associated elevated blood levels of thyroid-stimulating hormone (TSH) and thyroglobulin Ab with early onset of IRAE and prolonged survival, proposing IRAE as predictive markers for clinical outcome [80].

Few if any preclinical reports link TRs to cancer immunotherapy. Nevertheless, TRs are implicated in the management of stress responses (e.g., during fibrosis), a precondition to malignant diseases. In this context, iodothyronine deiodinase 2 (DIO2), the enzyme which converts and activates thyroxine (T4) to 3,3′,5-triiodothyronine (T3) [81] was elevated in patients with lung fibrosis, and *Dio2* knockout mice suffered from a more severe disease. Sobetirome, a synthetic mimetic or delivery of thyroid hormone as an aerosol, mitigated lung fibrosis in mice. Mechanistically, TR agonists improved the biogenesis and function of mitochondria in alveolar epithelial cells. T3-stimulated [82] murine DCs switched to an IL17+IFNγ+ pro-inflammatory phenotype and down-regulated PDL1/2 expression on CD4+ γδ T-cells in vivo. In parallel, FOXP3+ Treg counts were decreased. 

Thus, TRs may represent novel checkpoints at the interface of endocrine and immune systems to modulate inflammatory responses and preclude cancer formation. However, direct TR antagonists have not yet been approved for cancer therapy, as opposed to inhibitors of upstream hormone-producing enzymes (carbimazole, methimazole) for the treatment of hyperthyroidism.

Inherited genomic and de novo mutations in the *THRA*/*B* genes [83] confer whole-body hormone resistance, and, as for other steroid receptors, serine phosphorylation alters the subcellular location and transcriptional activity of the NHR [84].

Many effects of thyroid hormones are also mediated via non-genomic mechanisms. T4 analog tetra-iodothyroacetic acid (tetrac) covalently bound to nanoparticles (Nano-diamino-tetrac, NDAT, Nanotetrac) targets the extracellular domain of integrin αvβ3 and inhibits PI3K and MAPK signal transduction in cancer cells [85]. T4 increased, whereas NDAT decreased *PDL1* mRNA and protein in human breast and colon cancer cells, thereby employing non-genomic activation of ERK1/2. These data proposed antagonism of T4-induced PDL1 expression as an immunostimulant for future cancer therapies. Moreover, NDAT [86] inhibited PI3K activation as well as PDL1 accumulation and proliferation in gefitinib-resistant colorectal cancer cells (Colo160224). NDAT also reduced PDL1 levels and tumor growth in HCT116 (*KRAS* mutant) xenograft mouse models.

These findings were confirmed by others [87] who found that T4 binds to integrin αvβ3 which in turn stimulates proliferation and PDL1 expression in oral cancer cells. Therein, nuclear translocation of PDL1, p300, ERK1/2, STAT3 and β-catenin was observed. However, untangling this complex crosstalk remains a challenge. More evidence for the subcellular distribution of PDL1 was collected in *BRAF* V600E-mutated colorectal cancer cells and tissues [88], where it accumulated in the nucleus to stimulate proliferation. Mechanistically, the transport of PDL1 protein to the nucleus took place by binding to phosphorylated ERK1/2. Nuclear PDL1 then up-regulated the cell cycle regulator BUB1 via interaction with thyroid hormone receptor-associated protein 3 (THRAP3), unraveling signaling nodules potentially drugable by thyroid hormones.

For patients with thyroid cancer, therapies with MAPK inhibitors (MAPKi) have been implemented, although with limited success [89]. The *BRAFV600E* mutation promotes the formation of an immunosuppressive tumor microenvironment by infiltration of MDSCs via a TBX3-TLR2-NFκB-CXCR2-MDSC axis, which, when inhibited, sensitized experimental mice to MAPKi therapy.

In homograft mouse models [90] of thyroid cancer, glioma and breast cancer, thyroid-stimulating hormone (TSH) augmented proliferation, invasion and PDL1 expression via the TSHR-PKA-JNK-cJun pathway. In contrast, TSH receptor inhibition in combination with PD1 Ab reversed immune evasion by activation of Teffs and attenuation of PDL1 expression in tumors and monocyte-derived DCs, the main producers of TSH in the tumor tissue microenvironment.

Thus, interference with endocrine axes upstream of NHRs adds to the therapeutic modalities for cancer immunotherapies. Again, membrane-bound receptors for rapid non-genomic effects of thyroid hormones may constitute integrins and TR variants, with their impact on cancer immunotherapies still to be validated [91]. For example, the germline mutation variant TRβPV elicits follicular thyroid carcinoma in mice via direct interaction with oncogenic driver proteins (PI3Kp85α, β-catenin).

### 3.7. Vitamin D Receptor (VDR)

Calcitriol, the active form of vitamin D (1α,25(OH)2D3), binds the VDR and regulates the transport of minerals (e.g., calcium, phosphate) in tissues of the skeleton, intestines and kidneys. As for TRs, mutations in the *VDR* gene underlie systemic hormone resistance pathologies (e.g., rickets). Food supplementations are expected to reinvigorate the host immune system against infections, though mechanistic and clinical evidence is still lacking [92].

For example, vitamin D [93], its upstream activating enzyme CYP27B1 and VDR induce transcription factors (STAT3, BACH2, c-JUN) in CD4+ T-cells to reduce IFNγ and increase IL10 expression in patients with severe COVID-19 [94]. Thereby, a transition from pro-inflammatory Th1 cells to anti-inflammatory IL10+ T-cells was enforced. Responses to immune checkpoint therapies are also influenced by gut microbiota, linking nutrition-driven vitamin receptors and their mutational variants to clinical success [95].

A prospective clinical non-interventional analysis revealed that vitamin D3 deficiency predicts poor clinical outcomes in patients with metastatic melanoma under treatment with immune checkpoint Abs (PD1/CTLA4), BRAF or MEK1/2 inhibitors [96] and resulted in shortened overall and progression-free survival with higher tumor burden and IRAE. In contrast, under normal serum hormone concentrations, these effects were reversed.

Transcriptomes collected from patients with melanoma revealed that [97] high VDR correlated with reduced cancer-related death in primary and metastatic disease and up-regulation of pathways conferring anti-tumor immunity. VDR+ tumors harbored high T-cell infiltration counts and low Wnt/β-catenin signaling. Vitamin D deficiency shortened survival in melanoma-bearing mice, while VDR overexpression mitigated metastasis. VDR signaling also inhibited Wnt/β-catenin-dependent gene expression in vitro. Providing first causal evidence, further clinical and functional studies are necessary to assess if VDR is a suitable target for boosting immunotherapies against cancer. 

In mice, pancreatic cancer stellate cells cause refractory therapy responses, but were deactivated by pH-buffering micelles that block autophagy and encapsulate VDR ligand (calcipotriol). In combination with PD1 Ab and chemotherapy, the desmoplastic, fibrotic and immunosuppressive tumor microenvironment was reshaped towards anti-tumor attack and improved animal survival [98].

A prospective study [99] in patients with non-small-cell lung cancer who received docetaxel in combination with rocaltrol revealed that low serum vitamin correlated with high genomic expression of inhibitory immune checkpoints (PD1, TIGIT, TIM3), but low presence of the costimulatory CD28 molecule on CD8+ and Vγ9Vδ2+ T-cells. 1α,25(OH)2D3 triggered nuclear translocation of VDR and epigenetic repression through methylation of the promoter regions of the *Pdcd1*, *Tim3* and *Tigit* genes, but activation of the *Cd28* gene by histone acetylation. It also stimulated the synthesis of Th1 cytokines and anti-tumor immunity via T-cell receptor activation in CD8+ and Vγ9Vδ2+ T-cells, as validated by CRISPR/Cas9 VDR knockout and overexpression, respectively. Notably, oral supplementation of the hormone recapitulated these in vitro effects in patients with non-small-cell lung cancer.

These direct genomic actions of VDR were contrasted by vitamin D response elements in the human *PDL1*/*PDL2* genes [100], which were bound by 1α,25(OH)2D3-ligated VDR, resulting in transcriptional up-regulation in epithelial or myeloid cells. Hormone-treated epithelial cells had blunted activation of primary human CD4+ and CD8+ T-cells and synthesis of inflammatory cytokines, an effect which could be reversed by PD1-blocking Ab. Species specificity was a confounding variable, in that vitamin D response elements were present in primates but not in mice, thus warranting caution for translational models regarding the utility of VDR as a target for cancer immunotherapy.

Consistently, 1α,25(OH)2D3 [101] diminished the release of pro-inflammatory cytokines (IFNγ, IL17, IL21), but increased expression of CTLA4 and the Treg marker FOXP3 on human CD4+CD25- T-cells, resulting in reduced IL2-dependent proliferation.

Thus, hormonal vitamin D acted as an anti-inflammatory agent and potential inducer of adaptive Tregs, features which may foster immunosuppression undesirable for effective host responses against cancer.

Similar to all endocrine receptors, post-translational modification (e.g., by serine phosphorylation [102,103], reviewed in [104]) and non-genomic actions of calcitriol and its precursor metabolite calcifediol have been uncovered [105]. The latter again showcased plasma membrane-associated VDR proteins [7,8]. However, their function and relation to cancer immunotherapies have to be deciphered. 

## 4. Metabolic Receptors

### 4.1. Peroxisome Proliferator-Activated Receptor Alpha (PPARA)

For decades, fibrates have been prescribed as lipid-lowering agents for metabolic disorders, mainly hypercholesterolemia to prevent atherosclerosis and cardiovascular disease (e.g., stroke, myocardial infarction), in part by altering the ratio of serum lipoprotein subsets [106].

Recently, fibrates as other NHRSF ligands gained attention as “sensitizer” compounds to overcome resistance or non-response to cancer immunotherapies.

A landmark clinical study revealed that caloric restriction [107] has a beneficial impact on chronic inflammatory and autoimmune diseases without impairing acute host defense. Here, fasting reduced the number and activity of circulating monocytes in humans and mice. Mechanistically, hepatic AMPK inhibited systemic CCL2 production via PPARα to lower mobilization of monocytes from the bone marrow.

A recent phase 1 trial (UMIN000017854) tested PD1 Ab (nivolumab) in combination with bezafibrate, a ligand for PGC1α coactivator-bound PPARα which already acted synergistically with PD1 blockage in mice, in patients with advanced non-small-cell lung cancer [108]. Herein, cytotoxic T-cell functions were enhanced via increased fatty acid oxidation in mitochondria, allowing more durable and effective anti-tumor responses.

Melanoma-bearing mice exposed to [109] fenofibrate and a recombinant chimpanzee-derived replication-defective adenoviral vector expressing T-cell epitopes of melanoma-associated antigens (AdC68-gDMelapoly) exhibited ameliorated metabolic stress symptoms and delayed tumor progression by vaccine-induced circulating and tumor-infiltrating CD8+ T-cells. Again, fatty acid oxidation rose, and more free glucose was available for functional T-cells. Thus, metabolic reprogramming of the tumor microenvironment gives a prospect for the repurposing of established drugs to empower cancer vaccine efficacies. 

In murine melanoma, bezafibrate activated mitochondrial metabolism [110] within cytotoxic T-cells by the up-regulation of fatty acid oxidation, oxidative phosphorylation and respiratory capacity. Mechanistically, CPT1 and BCL2 proteins formed a complex to prevent cellular apoptosis, thereby maintaining prolonged survival of cytotoxic T-cells under checkpoint therapy.

In a murine model of adoptive cell therapy, the dual PPARα/δ agonist GW501516 enhanced the expression of *CPT1A*, the rate-limiting enzyme of fatty acid oxidation in activated T-bet+ CD8+ T-cells [111], accompanied by a release of pro-inflammatory mediators from DCs (IL12, IFNγ). Notably, ligand-treated CD8+ T-cells displayed a memory phenotype suggesting long-term in vivo persistence compared to short-lived effector cells, encouraging translation into humans.

Similar results were obtained from mice with melanoma [112] exposed to hypoglycemia and hypoxia, which up-regulated PPARα-dependent fatty acid oxidation, a metabolic switch that preserved intra-tumoral CD8+ Teff functions and attenuated tumor progression in conjunction with PD1 blockade.

Similar to PPARα/δ agonists, precursors of reactive oxygen species, AMPK activators and mitochondrial uncouplers [113] potentiated the tumoricidal activity of PD1 Abs by expansion of mitochondrial mass and effector/memory cytotoxic T-cells in draining lymph nodes and within the tumor. This effect was marked by increased levels of mTOR, AMPK, PGC1α and T-bet proteins. Thus, metabolic reprogramming adds a stepstone towards combinatorial immunotherapies.

Obesity has been linked to altered sensitivity to checkpoint immunotherapies; however, experimental evidence for causal mechanisms is limited [114]. Melanoma-bearing obese mice on a high-fat diet suffered from massive lipid accumulation in NK cells, impairing their effector functions against tumors. Mechanistically, PPARα/δ agonists inhibited mTOR-mediated glycolysis, thereby precluding the formation of the “synapse” between NK and tumor cells. Vice versa, inhibition of PPARs or mitochondrial lipid transport alleviated NK dysfunction and restored cytotoxicity.

Non-invasive glucose imaging showcased that tumor-associated macrophages [115] promoted hypoxia and aerobic glycolysis in mice with subcutaneous xenografts and in patients with non-small-cell lung cancer. Therein, TNFα, AMPK and PGC1α were the major factors responsible for this metabolic program. This phenotype could be reversed by the depletion of tumor-associated macrophages with clodronate and allowed improved anti-tumor response to PDL1-blocking Abs upon up-regulation of PDL1 on tumor cells and increased infiltration by T-cells.

Conclusively, innate and adaptive immune cells can be metabolically fine-tuned by PPAR-driven oxidative and glycolytic pathways to benefit immunotherapy success.

Direct genomic actions of PPARα confer protection against cardiotoxicity, a serious IRAE during checkpoint therapy [116]. As such, mice treated with BMS1, a low molecular inhibitor of PD1-PDL1 protein interactions, suffered from intestinal barrier injury and gut microbiota dysbiosis, as evidenced by the depletion of *Prevotellaceae* and *Rikenellaceae* vs. the gain of *Escherichia-Shigella* and *Ruminococcaceae*. This imbalance promoted M1 polarization of colonic macrophages and synthesis of pro-inflammatory cytokines (TNFα, IL1β) via the down-regulation of PPARα-dependent *CYP4X*1 transcription, a PPAR-ligand-generating enzyme. Fecal microbiota transplantation mimicked the cardiotoxic effect of BMS1, whereas *Prevotella loescheii*, butyrate, macrophage depletion and TNFα/IL1β neutralization mitigated cardiomyocyte apoptosis. Thus, targeting PPARα may help to avoid lethal adverse events of checkpoint Abs.

In mice with lung carcinoma xenografts [117], bezafibrate and PDL1 Ab achieved synergistic anti-tumor efficacy. PPARα agonist induced chemokine expression in the tumor (CXCL9/10) together with the respective receptor (CXCR3) on CD8+ T-cells, allowing their recruitment into the tumor tissue microenvironment. These events were accompanied by enhanced survival and functionality, as given by the accumulation of radicals and a gene signature for fatty acid oxidation (*PGC1A*, *CPT1A*, *LCAD*), proposing PPARα/PGC1α as a potential adjuvant to immune checkpoint therapy.

Despite ample evidence for functional regulation of all three PPAR proteins through post-translational modifications, exemplified by phosphorylation, sumoylation, ubiquitination, acetylation and glycosylation [118], the non-genomic crosstalk of PPARα with MAPKs in relation to immune checkpoints has yet to be explored. 

Several plasma membrane-associated non-NHR receptors have been examined to explain the rapid non-genomic signaling effects of physiological and synthetic PPAR ligands on MAPKs. Those include GPCRs of the “Free Fatty Acid Receptor” (FFAR) family [119] or the EGFR [120]. In contrast to endocrine NHRs, specific membranal PPAR variants have not been described. Instead, PPARα activators [121] directly induce genes encoding for xenobiotic-metabolizing enzymes (e.g., cytochrome P450) and simultaneously act as competitive enzyme inhibitors to maintain homeostatic concentrations of endogenous ligands and xenobiotics. As such, PPARα/β/γGPR40 pan-agonist RLA8A was designed to ameliorate non-alcoholic steatohepatitis and fibrosis in mice [122], leaving the answer open if this quadruple targeting may also counteract cancer development.

### 4.2. Peroxisome Proliferator-Activated Receptor Gamma (PPARG)

Long-term intake of potent and selective PPARγ agonists, anti-diabetic insulin sensitizers of the glitazone or glitazar classes, has been limited due to pleiomorphic side effects on many organs such as bone fractures, weight gain, edema and heart failure, resulting in partial retraction from the market worldwide [123]. Unfortunately, this is not a compound class effect but causal to the adipogenic receptor. Nonetheless, the repurposing of PPARγ agonists of all classes has evinced potential in combination with chemo- and targeted therapies against tumors and cardiovascular and metabolic diseases beyond type 2 diabetes.

A recent landmark phase 2b clinical trial demonstrated the clinical benefit of pan-PPARα/γ/δ agonist lanifibranor in non-alcoholic steatohepatitis to slow the progression of fibrosis (NCT03008070) [124]. A multicenter study (NCT00091949) showed protection against future cardiovascular events after ischemic stroke by PPARγ agonist pioglitazone [125]. In view of repurposing, pioglitazone was found to erode the quiescent cancer stem cell pool in patients with chronic myeloid leukemia [126] via decreased expression of the stemness markers STAT5, HIF2α and CITED2. 

In line with these data, preclinical inhibition of PPARγ in cells of the myeloid lineage induced systemic inflammation, immunosuppression and tumor formation [127]. Transgenic mice with dominant-negative PPARγ in myeloid cells had bone marrow progenitor populations enriched with STAT3, NFκB and MAPKs (ERK1/2, p38), pro-inflammatory cytokines (IL1β, IL6, TNFα) and high MDSC counts. The latter inhibited proliferation and cytokine synthesis in CD4+ and CD8+ T-cells, resulting in the formation of carcinomas and sarcomas in multiple organs.

Preclinical studies explored whether obesity in conjunction with predictive microbiome signatures relates to breast cancer outcomes. Therein, PD1 Ab mitigated tumor progression in lean mice to a higher extent than in obese ones, despite augmented tumor burden under obese conditions [128]. This observation alluded to a crosstalk of adipogenic PPARγ signaling and response to checkpoint inhibition.

Similar experimental evidence [129] connected obesity to treatment response in mouse models of inflammation (atopic dermatitis). Obesity converted Th2+ towards a severe Th17+ disease. PPARγ was reduced in Th2 cells from obese mice compared to lean littermates, but was necessary for a functional Th2 response and the prevention of non-Th2 severe inflammation. PPARγ agonist (rosiglitazone) mitigated Th17 pathology and sensitized animals to anti-Th2 IL4/IL13-neutralizing Abs. 

A study uncovered that PPARγ [130] stimulated anti-tumor immunity under GM-CSF-secreting tumor cell vaccines. Myeloid-specific *Pparg* knockout reduced the ratio of CD8+ Teffs in favor of Treg cells due to DC-mediated release of chemoattractants (CCL17, CLL22). In contrast, PPARγ agonists restored CD8+ Teffs pools, potentiated vaccine-driven anti-tumor response in combination with CTLA4 Ab in vivo, and reduced chemokine expression in tumor cell co-cultures with human peripheral blood mononuclear cells in vitro.

Supporting this immune sensitizer role, PPARγ [131], together with the transcription factors PLZF and SREBF1, induced lipid biosynthesis (e.g., cholesterol) in invariant natural killer T (iNKT) cells to amplify IFNγ release. In contrast, lactic acid in the tumor microenvironment lowered PPARγ levels in iNKT cells, abrogating IFNγ production. PPARγ agonist (pioglitazone) restored IFNγ production in tumor-infiltrating iNKT cells from patients and mice, and in combination with an immunostimulant (α-galactosylceramide), enforced iNKT cell-driven anti-tumor responses for prolonged animal survival.

Overall, the immune-regulatory function of PPARγ and its ligands may be useful to fine-tune immune responses in tumor-bearing hosts. However, its role remains controversial.

As such, PPARγ [132] drives pro-tumoral functions of group 2 innate lymphoid cells (ILC2) which protect against infection by helminths via the release of IL33, an alarmin overexpressed in cancers. Pharmacologic inhibition or genetic deletion of PPARγ in ILC2s abolished IL33-induced Th2 cytokine production and tumor growth in vivo, suggesting PPARγ inhibition as a target for ILC2-driven diseases beyond cancer (e.g., asthma, allergy).

Likewise, evasion of immunosurveillance by constitutive activation of *PPARG*/*RXRA* genes was noted in muscle-invasive bladder cancer [133]. Clinical data and mouse tumor models showed that high PPARγ/RXRαS427F/Y positivity predicts low infiltration of CD8+ T-cells and imposes resistance to immunotherapies. Instead, PPARγ/RXRα knockdown or pharmacological inhibition restored pro-inflammatory cytokine production, alluding to a tumor-specific role of this NHR warranting the careful choice of agonists vs. antagonists to achieve clinical benefit.

T-cells deficient for sphingosine-kinase-1 [134] exhibit higher mitochondrial respiration and reduced differentiation to a regulatory immunosuppressive phenotype. Since this kinase positively correlated with PPARγ activity, and as its genetic and pharmacologic inhibition together with PD1-blocking Ab improved metabolic fitness and anti-tumor activity of T-cells against murine melanoma, PPARγ antagonists may be envisioned as agents to improve tumor control.

Direct genomic actions of PPARγ on immune checkpoint genes have been investigated. ILC2 cells from PD1−/− RAG1−/− mice failed to produce cytokines protective against helminth infection (IL5, IL13) and allergy [135]. Notably, PD1 on ILC2 was increased by PPARγ agonist and decreased by PPARγ antagonist, suggesting that it is a PPARγ target gene.

Consistently, rosiglitazone [136], in combination with IL5-neutralizing Ab, prevented MHC class II mismatched graft rejection in mice by reductions in vasculopathy, collagen load and infiltration of CD8+ T-cells and eosinophils. Notably, graft survival correlated with increased expression of PDL1 in the transplanted tissue.

Thus, PPARγ agonism may enhance the immunogenicity of otherwise “cold” tissues, a feature allowing improved drug-target engagement, e.g., by ligation of therapeutic PD/PDL1-blocking Abs to their epitopes on the respective counter molecule.

Despite the long history of PPARγ agonists in the treatment of diabetics, how non-genomic effects of receptor phosphorylation (e.g., by MAPK, CDKs, CKs, etc.) impact the clinical efficacy of these drugs is still under investigation [137,138]. Therein, antagonistic and synergistic effects of this post-translational modification have been reported, especially in the context of ERK1/2 [139].

On the contrary, knowledge of the interaction of PPARγ with MAPK pathways in view of immunotherapy or related biologicals is limited [140]. In mice on a high-fat diet, the oncogenic *KrasG12D* mutant promoted the formation of invasive pancreatic ductal adenocarcinoma. In acinar cells, *KrasG12D* reduced *Fgf21* mRNA due to the down-regulation of PPARγ, a positive regulator of this gene. Injection of recombinant FGF21 inhibited RAS and attenuated inflammation-driven tumor formation, proposing intervention at the RAS-PPARγ-FGF21 axis as a novel target to treat or prevent pancreatic cancer.

In human keratinocytes of basal and squamous cell carcinomas [141], the surface receptor CD200 triggered the loss of NK cells. Mechanistically, MMP3/11 cleaved the CD200 ectodomain, followed by shedding the soluble fragment into the tissue, where it bound to NK cells. Therein, MAPK signaling, IFNγ production and cytotoxicity were inhibited via reduced ERK1/2-driven inhibitory phosphorylation of PPARγ and up-regulation of pro-apoptotic PPARγ target genes (FasL, Fas/FADD). Vice versa, CD200 blockage or PPARγ inhibition restored the survival and killing capacities of NK cells, proposing the CD200-ERK1/2-PPARγ axis as a potential novel immune checkpoint.

Later reports claimed that PPARγ agonists of the glitazone class directly transactivate the EGFR, leading to its dimerization, p60Src kinase-dependent tyrosine phosphorylation and downstream signaling. Mechanistically, this was supposed to involve calcium ion channels at the endoplasmic reticulum or mitochondria [120].

As is true for all three PPARs, non-genomic plasma membrane receptors for physiological free fatty acids and the glitazone class of synthetic PPARγ agonists have been validated to rapidly transduce signals towards the cell interior and activate MAPK signaling, proliferation and survival of cancer cells [142]. Nonetheless, their specific role in cancer immunotherapy is unknown. 

Free fatty acid receptors (GPR40 (FFAR1), GPR43 (FFAR2), GPR41 (FFAR3), GPR120 (FFAR4), etc.) [119] were originally developed as anti-diabetic targets, albeit with landmark insight into their utility for the treatment of inflammatory diseases. In mice, GPR40 agonist (GW9508) stimulates the function of neutrophils to eliminate infectious pathogens (e.g., *Escherichia coli*) [143] via increased IL8-guided chemotaxis, phagocytosis and resolvin production. Natural ligands (e.g., palmitic acid esters of hydroxy stearic acids) through GPR40/GLP1R attenuate detrimental organ infiltration of B/T-cells and promote pancreatic β-cell survival in type 1 diabetic mice [144] by reducing endoplasmic reticulum stress and ERK1/2-JNK1/2 signaling. 

Omega-3 fatty acids (eicosapentaenoic and docosahexaenoic acid) bind to GPR120/GPR40 in macrophages and mitigate inflammation through the inhibition of the NLRP3 inflammasome, caspase-1 and IL1β secretion [145] in type 2 diabetic mice.

PPARγ agonist (rosiglitazone) [146] also cooperated with GPR120 agonist to dampen diabetes in mice. Here, GPR120 was identified as a PPARγ target gene and, vice versa, GPR120 counteracted ERK1/2-mediated inhibition of PPARγ in macrophages and adipocytes. 

Likewise, GPR120 agonist ameliorated colitis through CD4+ T-cell-mediated IL10 synthesis in *Citrobacter rodentium*-infected mice [147]. GPR40 also acted anti-inflammatory in macrophages of mice with high-fat-diet-induced hepatic steatosis and fibrosis [148]. 

Finally, platinum-induced fatty acid 16:4(n-3)(hexadeca-4,7,10,13-tetraenoic acid) triggered GPR120-mediated signaling in macrophages to promote resistance to DNA-damaging chemotherapy via cPLA2-mediated synthesis of chemoprotective lipids [149], collectively alluding at a yet unknown potential of free fatty acid receptor intervention to improve anti-tumor responses also in humans.

### 4.3. Retinoic Acid Receptors (RARs)

Vitamin A and its derivatives (e.g., ATRA, all-trans retinoic acid) are valued as food supplements in systemic applications and as topical remedies for skin diseases (acne, psoriasis). ATRA enjoys a long history of medication in patients with acute promyelocytic/myeloid leukemia, although its relation to immunotherapies has yet to be elucidated. Three genes encode for RARs (α/β/γ). 

A landmark clinical study reported that ATRA leads to the eradication of acute promyelocytic leukemia-initiating cells by the degradation of PML-RARα fusion protein [150]. This agent also induced differentiation of human acute promyelocytic leukemia cells and clearance of murine malignant PML-RARα+ cells in vivo.

A phase 1/2 clinical trial combined PD1 Ab (pembrolizumab) and ATRA for the treatment of metastatic melanoma [151] and confirmed anti-tumor activity, as evidenced by the reduction in circulating MDSCs and prolonged progression-free survival. Mechanistically, ATRA targeted MDSC differentiation, squelching the tumor microenvironment into a permissive state for checkpoint therapy.

Recent translational and preclinical research has shed light on ATRA [152] for boosting host immunity. For example, human mesenchymal stromal cells induce the differentiation of human monocytes into CTLA4+ DCs via the production of the RARα agonist ATRA. RARα is critical for DCs to activate allogeneic, naive T-cells to differentiate into IL10+IL17+ Th cells in a CTLA4-dependent manner. These data underscored the importance of components of the tumor microenvironment in shaping immunoprofiles.

Intriguing combination therapies demonstrated that blockade of the myeloid checkpoint CD38 by daratumumab, a therapeutic Ab approved for multiple myeloma, invigorated the killing of acute lymphoblastic T-cell leukemia cells in vitro [153]. Notably, antibody-dependent cellular phagocytosis (ADCP) by macrophages and antibody-dependent cell-mediated cytotoxicity (ADCC) by granulocytes was more efficient with IgA than IgG Abs and inhibited by SIRPα/CD47 (“do-not-eat-me”) interaction with tumor cells. ATRA increased CD38 surface expression, leading to enhanced ADCP and ADCC of leukemic cells in the presence of CD38 or CD47 (magrolimab) blocking Ab. 

Consistently, the inhibition of granulocytic MDSCs [154] overcame *KRAS*/*LKB1* mutation-dependent resistance to immune checkpoint therapy in cells and genetic mouse models of non-small-cell lung cancer. Deletion of LKB1 triggered the release of CXC-type chemokines in vitro and in vivo to recruit and elevate G-MDSCs in the local tumor tissue microenvironment, spleen and blood circulation. G-MDSC depletion with Abs or ATRA enforced anti-tumor T-cell responses and sensitized murine tumors to PD1 Ab, proposing this combination for the reversal of MDSC-driven immunosuppression also for patients.

Accordingly, functional inactivation of highly prevalent PDL1+ MDSCs with ATRA enhanced PDL1 Ab efficacy in patients with cervical cancer [155]. In mice xenografted with cervical tumors and treated with ATRA and PDL1 Ab, MDSCs were diminished and tumor growth decreased, presumably mediated by high intra-tumoral infiltration of CD4+ and CD107a+CD8+ cytotoxic T-cells and the production of pro-inflammatory cytokines (IFNγ, TNFα).

ATRA also overcomes resistance to radiotherapy by strengthening abscopal (i.e., regression) effects distal from the site of irradiation [156]. In syngeneic mouse models of colon cancer, ATRA, together with ionizing radiation or PDL1-blocking Ab, improved anti-tumor control compared with the respective monotherapies, as evidenced by a massive increase in pro-inflammatory TNFα+ iNOS+ macrophages and IFNγ+ CD4+ and CD8+ T-cells in the local tumor tissue and systemically.

Moreover, RAR agonists exert genomic actions on immune checkpoint genes. For example, ATRA up-regulated PDL1 in gastric cancer cells and impaired cancer immune surveillance in vivo [157]. Mechanistically, the synthesis and stability of PDL1 protein increased in an IFNγ-JAK-dependent manner, conferring resistance to T-cell-driven killing. Resistance was reversed by JAK-inhibitor (ruxolitinib), which resensitized gastric cancer cells to PDL1 Ab.

Maturation of human myeloid blasts by IFNγ in combination with ATRA or 1α,25-dihydroxyvitamin D3 [158] increased the expression of CD11b, PDL1/2 and STAT3, a major pro-leukemogenic transcription factor in acute myeloid leukemia and myelodysplastic syndrome. STAT3 inhibitor (stattic) reversed PDL1/2 induction but maintained IFNγ-mediated anti-tumor responsiveness, underscoring that immune evasion can be alleviated. 

In human eosinophils [159], ATRA and 9-cis-retinoic acid favored cell survival and nuclear hypersegmentation similar to IL5 in an RAR/RXR-dependent manner. This was accompanied by the secretion of VEGFA, CSF1/M-CSF and MCP1 and the down-regulation of caspase-3, proposing an anti-apoptotic role for vitamin A derivatives, not only in allergic conditions but also in the tumor tissue microenvironment, implying a potential use for RAR agonists as immunomodulators.

Non-genomic interactions with kinases were reported. In oral squamous cell carcinoma cell lines [160], ATRA induced cell cycle arrest and apoptosis via altered phosphorylation (STAT3, JAK2, ERK1/2) and expression (PDL1) of target proteins/genes. Again, a small-molecule STAT3 inhibitor potentiated the growth inhibitory effect of ATRA, blunted PDL1 expression and elevated cleaved caspase-3. 

ATRA [161], together with tyrosine kinase inhibitor (midostaurin), triggered apoptosis in FLT3 wt or mutant acute myeloid leukemia cells and exerted anti-tumor efficacy in mouse xenograft models. Mechanistically, this drug combination activated caspase-3/7 and inhibited Lyn/Fgr/Hck-dependent AKT phosphorylation, resulting in RAF-MEK1/2-ERK1/2 activation and up-regulation of lineage-determining transcription factors (C/EBPβ, C/EBPε, PU.1.), proposing this regimen as a potential future myeloid differentiation therapy.

As for PPARs, phosphorylation by MAPK impacts the localization and activity of this NHR [162]. However, specific non-genomic receptors (non-NHR) at the plasma membrane have not been discovered; instead, retinoids can bind to intracellular RARα pools in lipid rafts or to cytosolic proteins (e.g., CRABPs [163]). In embryonic and neuronal stem cells, CRABP1 dampens growth factor sensitivity and stemness [164]. CRABP2 is required for the synthesis and signaling of ATRA in human and mouse DCs [165] affecting gut mucosal immunity, suggesting additional crosstalk of NHR and non-NHR pathways for vitamin A derivatives.

### 4.4. Retinoid X Receptors (RXRs)

RXRs (α/β/γ) are obligate heterodimerization partners of adopted orphan receptors of the non-endocrine NHRSF subclass. Therefore, the physiological ligand 9-cis retinoid acid and drug derivatives (termed rexinoids) have the potential to redirect NHRSF-transcriptomes dependent on the respective heterodimer partner.

9-cis-retinoic acid (alitretinoin) is prescribed for the topical treatment of skin disease (AIDS Kaposi’s sarcoma, eczema). Bexarotene is currently the only rexinoid approved for clinical use. A phase 3 clinical study [166] confirmed improved response rates and progression-free survival with bexarotene-proficient multimodal chemotherapies in patients with cutaneous T-cell lymphoma [167].

Regarding benefits in cancer immunotherapy, preclinical reports claim that the selective RXR agonist LG100268 modulates the immune microenvironment in murine breast cancer [168]. LG268, but not bexarotene, lowered infiltration of MDSCs and CD206+ macrophages, resulting in a rise in PDL1 positivity and CD8+ vs. CD4+CD25+ T-cell ratios and cytotoxicity against Her2+MMTV-Neu and triple-negative MMTV-PyM-driven breast cancer. Thereby, LG268 sensitized mice to the anti-tumor efficacy of PDL1-blocking Ab. 

RXR [169] also promotes the expansion of mouse tissue-resident macrophages of the serous cavity to foster the progression of ovarian cancer. In contrast, the absence of RXR reduces the neonatal progenitor pool and survival of adult macrophages and mitigates tumor progression. Mechanistically, RXR deficiency, via chromatin remodeling and transcriptional reprogramming of canonical macrophage genes, evoked lipid overload in the tumor, suggesting RXR antagonists for therapy.

In mice, repurposing of an agonist specific for RXR/RXR homodimers (IRX4204) attenuated graft-versus-host disease to strengthen graft-versus-leukemia response [170] by elevating the ratio of tolerogenic Tregs vs. Th1 cells and restricting the proliferation of allogenic donor T-cells. IRX4204 lowered the transcription of pro-inflammatory cytokines (IFNγ, TNFα) in vivo and in vitro, proposing the use of rexinoids as immune modulators in the clinics.

Direct genomic actions of RXR on immune checkpoints have been uncovered. In human triple-negative breast cancer cells, the transcription factor EGR1 and RXRα bind the promoter and transcriptionally induce the macrophage-attracting chemokine *CCL2* upon stimulation with TGFβ [171], a pivotal cytokine involved in epithelial-to-mesenchymal transition, a hallmark of cancer progression and metastasis. 

In human-monocyte-derived macrophages [172], IL4-induced STAT6 in part colocalized with RXR within the proximal genomic cistrome and at distal gene-selective enhancers, suggesting a functional crosstalk of RXR with STAT6 at DNA-binding motifs determining the polarization status of macrophage subsets.

As before, serine-directed phosphorylation events also govern RXR activities and subcellular localizations [173], specifically via PI3K/AKT [174], ERK1/2 [175] and JNK [176,177].

MAPK-specific non-genomic RXR crosstalk has been demonstrated in HER2+ and KRAS-driven cancer models. In immune-competent mice of Her2+MMTV-Neu breast cancer and chemical-induced lung cancer, RXR agonist (MSU42011) [178] attenuated tumor growth in combination with PD1/PDL1-blocking Abs. This effect was achieved by an increased CD8+ to CD4+CD25+ T-cell ratio, whereas it failed in athymic human lung cancer xenograft models, suggesting the requirement of mature T-cells to exert RXR-mediated inhibition of the oncogenic HER2-MAPK cascade.

Overall, through its ability to form homo- vs. heterodimers, targeting RXR may envision means to fine-tune downstream programs of individual NHRs. Extranuclear receptor variants or transmembrane receptors for rapid non-genomic effects of rexinoids or 9cis-retinoic acid have not been explored so far [179].

### 4.5. Farnesoid X Receptor (FXR)

The bile acid receptor FXR is primarily responsible for the homeostasis of bile acid metabolism in the liver and the intestines. It has been proposed as a target for the treatment of metabolic diseases based on its lipid-lowering, differentiation-promoting and anti-inflammatory abilities, justifying its notion as a potential tumor suppressor. 

The bona fide ligand ursodeoxycholic acid (UDCA) has been approved for clinical therapy of gall-bladder-related diseases (e.g., gallstones, cholestasis, cholangitis). Landmark trials confirmed clinical benefits for patients with primary biliary cholangitis and, eventually, cirrhosis [180,181]. Recently, the semi-synthetic agonist obeticholic acid (OCA) has been appreciated in phase 3 clinical trials to ameliorate fibrosis in patients with non-alcoholic steatohepatitis [182] and primary biliary cholangitis [183]. 

The potential of preclinical FXR modulation in cancer immunotherapies is just emerging [184]. In patients with non-small-cell lung cancer, FXR was inversely associated with PDL1 expression, and FXR overexpression down-regulated PDL1 via gene trans-repression in cells and synergized with PD1-blocking Ab in syngeneic lung cancer mouse models. However, CD8+ Teff functions and proliferation were diminished in co-cultures of FXR+PDL1- lung cancer cell lines, suggesting FXR as a biomarker for an immunosuppressive yet checkpoint-inhibitor-responsive tumor microenvironment.

Nanoparticles loaded with obeticholic acid were enriched in liver sinusoidal endothelial cells and Kupffer cells and attenuated tumor growth in an orthotopic mouse model [185], accompanied by increased secretion of CXCL16 and IFNγ and expansion of natural killer T (NKT) cell populations within the tumor. Thus, precision delivery of FXR agonists to selective target cells may open novel options for enforcing host immunity against liver cancer.

Direct genomic actions on immune checkpoints are exemplified by the loss of protective functions of intestinal FXR in inflammatory bowel disease [186]. In mouse colitis models, FXR activation controlled IL17 expression in innate lymphoid cells (ILCs) and led to the expansion, differentiation and maturation of ILC precursor-like cells, proposing FXR as a potential therapeutic target also for patients. 

In a similar study [187], FXR agonist obeticholic acid diminished the proliferation and metastasis of human hepatocellular carcinoma cells. Therein, STAT3 phosphorylation and JAK2, IL1B and IL6 expression were reduced, and bona fide FXR-target genes SOCS3 and SHP increased. FXR antagonist (guggulsterone) reversed the effects of obeticholic acid on cell cycle arrest, cytotoxicity, invasion and migration. Therefore, pharmacological FXR activation may be exploited to reprogram cytokine profiles in the tumor microenvironment. 

Consistently, FXR antagonist guggulsterone reduced cell viability and cell cycle progression in vitro and abrogated tumor growth in mouse lung carcinoma in vivo. This was achieved by transcriptional up-regulation of the *PDL1* gene via FXR inhibition and activation of AKT-ERK1/2 signaling in non-small-cell lung cancer cells [188].

In addition to post-translational modifications of FXR itself by phosphorylation [189], several non-genomic interactions with kinases have been uncovered. 

In human colorectal cell lines [190], FXR antagonist (guggulsterone) stimulated phosphorylation of the EGFR, p60Src and ERK1/2, whereas FXR agonist (GW4064) or overexpression reversed this effect and inhibited proliferation in vitro and in nude mice with human colon cancer xenografts. Likewise, inhibition of all three kinases abrogated tumor cell growth, suggesting a drugable crosstalk between the NHR and major oncogenic driver pathways in colorectal cancer.

Notably, physiologically relevant non-genomic actions of bile acids are transduced by TGR5, a GPCR at the plasma membrane [191]. However, if this receptor contributes to immune responses in cancer remains unclear. As such, UDCA in combination with PD1 Ab inhibited Treg differentiation and established anti-tumor immune memory in mice [192] and also synergized with PD1/PDL1 Abs in patients. Mechanistically, UDCA bound to TGR5 on the plasma membrane, triggering PKA-dependent phosphorylation of TGFβ followed by binding to Hsc70-interacting protein (CHIP), ubiquitination and degradation of TGFβ. By this non-genomic action, UDCA mitigated TGFβ-driven immunosuppression.

Secondary bile acids generated by gut microbiota ameliorated experimental autoimmune uveitis in mice via TGR5 [193]. Mechanistically, NFκB-driven expression of pro-inflammatory cytokines in DCs was prevented through the activation of PKA by TGR5 agonist. Bile acids also triggered phosphorylation and ubiquitination of the NLRP3 inflammasome via TGR5-cAMP-PKA in vivo [194]. 

TGR5 further reduced the number, activity and migration of macrophages via AKT-mTOR-C/EBPβ [195] in obese mice, and pharmacological TGR5 activation decreased endotoxin-induced expression of chemokines in primary macrophages. In contrast, TLR4 and TGR5 agonists (e.g., betulinic acid, CDCA, DCA) [196] coactivated MAPKs (ERK1/2, p38, JNK) and NFκB in human monocytic cells, triggering synergistic production of inflammatory cytokines, proposing them as a target to intervene with inflammatory host responses. 

Importantly, TGR5 activated the cAMP-STAT3/STAT6 axis to promote M2 polarization of tumor-associated macrophages and suppress CD8+ T-cell anti-tumor immunity in non-small-cell lung cancer mouse models [197]. Its expression correlated with poor patient prognosis, nominating TGR5 inhibitors for future cancer therapies.

## 5. Conclusions and Clinical Perspectives

The literature collected in this review highlighted the potential of genetic and pharmacological intervention at the level of RTK-MAPKs and the NHRSF to rewire host immune responses from an immunosuppressive towards a tumor-attacking mode. From our own research experience, the repurposing of NHR ligands (e.g., anti-diabetic PPARγ agonists) up-regulated PDL1 expression in human gastrointestinal cancer cell lines and PDOs and sensitized co-cultures of PDOs with cytotoxic CD8+ Teffs and NK cells to growth inhibition and cell death [198]. In *KRAS* mutant mice, we could show that rosiglitazone reprogrammed macrophages within the tumor tissue microenvironment to a pro-inflammatory M1-like phenotype [199,200], enabling tumor control. Overall, experimental evidence based on in vitro or ex vivo models together with preclinical data in rodents is convincing; however, systematic and randomized, double-blind, controlled clinical trials with extended patient numbers stratified for molecular cancer subtypes are necessary to fully reveal the potential future benefit of this approach. The intricate spatio-temporal network of MAPK-NHRSF crosstalk further demands in-depth mechanistic studies to understand the biology and risks of adverse events/side effects in order to optimize the clinical benefits.

## Figures and Tables

**Table 1:** Overview of nuclear hormone receptors in immunotherapies.

MOA	NHR	Ligand *	Type	Clinical Trials with Checkpoint Abs §
Immune activator	RARA	ATRA	+	NCT05482451, NCT04305041, NCT04305054, NCT05482451, et al.
	VDR	Rocaltrol	+	NCT03197636, NCT04615988, NCT03331562
	PPARG	Glitazones	+	NCT04114136, NCT02852083, NCT02767063
	RXR	Bexarotene	+	NCT01578499, NCT00030849
	PPARA	Fibrates	+	$
	FXR	UDCA, OCA	+	$
Immune suppressor	ER	Tamoxifen	−	NCT03725059, NCT02648477, NCT03147287, NCT02997995, et al.
	AR	Abiraterone	−	NCT04946370, NCT04191096, NCT03753243, NCT04116775, et al.
	PR	Mifepristone	−	NCT04046185, NCT03225547
	MR	Spironolactone	−	$
	GR	Dexamethasone	+	NCT02289222, NCT03834506, NCT03605719, NCT05096663, et al.
	TR	Sobetirome	+	$

* Selected bona fide ligand (Agonist = “+”; Antagonist = “-”); § Selected clinical trials (“URL accessed on 25 June 2023” https://clinicaltrials.gov) testing combinations of NHR ligands with immune checkpoint Abs (PD1, PDL1, CTLA4, et al.; interferon) in patients with cancer (solid tumors, leukemia, lymphoma, etc.), $ excluding trials with NHR ligand monotherapy or non-checkpoint combination regimens (chemotherapy, RTK blocking Abs, epigenetic/signaling inhibitors, etc.).

## Abbreviations

Abs, antibodies; AKT, protein kinase B; AMPK, AMP-activated protein kinase; AR, androgen receptor; CAR-T, chimeric antigen receptor T-cells; CDK, cyclin-dependent kinase; CK, casein kinase; CTLA4, cytotoxic T-lymphocyte-associated protein-4; DBD, DNA-binding domain; DC, dendritic cells; EGFR, epidermal growth factor receptor; ER, estrogen receptor; EAE, experimental autoimmune encephalomyelitis; ERK1/2, MAPK3/1; FXR, farnesoid X receptor; GPCR, G-protein coupled receptor; GR, glucocorticoid receptor; IRAE, immune-related adverse events; JNK/SAPK, MAPK8; LBD, ligand-binding domain; MAPK, mitogen-activated protein kinase; MEK, MAPK-kinase; MR, mineralocorticoid (aldosterone) receptor; mTORC, mammalian target of rapamycin complex; NHRSF, nuclear hormone receptor superfamily; NK, natural killer cells; p38, MAPK14; PD1, programmed cell death-1; PDL1, programmed cell death-1 ligand-1; PI3K, phosphoinositide-3-kinase; PKA/C, protein kinase A/C; PPAR, peroxisome proliferator-activated receptor; PR, progesterone receptor; RAF, rat rapidly accelerated fibrosarcoma viral oncogene homolog; RAR, retinoic acid receptor; RXR, retinoid X receptor; RAS, Kirsten rat sarcoma viral oncogene homolog; RTK, receptor tyrosine kinase; SRC, steroid/nuclear receptor coactivator; T/B, lymphocytes; Tc, cytotoxic T-cells; Teff, effector T-cells; Th, helper T-cells; TR, thyroid hormone receptor; Treg, regulatory T-cells; VDR, vitamin D receptor; wt, wildtype.
